# Editorial: How RSV outsmarts the host

**DOI:** 10.3389/fimmu.2024.1529014

**Published:** 2024-12-20

**Authors:** Stephania A. Cormier, Lawrence M. Kauvar, Ralph A. Tripp

**Affiliations:** ^1^ Pennington Biomedical Researcher Center and Department of Biological Sciences, Louisiana State University, Baton Rouge, LA, United States; ^2^ Division of Research, Trellis Bioscience, Inc., Redwood City, CA, United States; ^3^ Department of Infectious Diseases, University of Georgia, Athens, GA, United States

**Keywords:** RSV, host response, immune response, treatment, vaccine

Respiratory syncytial virus (RSV) is a respiratory pathogen that causes respiratory tract disease in approximately 64 million people worldwide and results in 160,000 deaths annually (1). Those at highest risk for severe disease are the most vulnerable populations, including premature infants, infants less than 6 months of age, older adults, and those with underlying cardiopulmonary disease or weakened immune systems. Recent advances have resulted in clinical options to offer high-risk patients: AREXY^®^, mRESVIA^®^, and ABRYSVO^®^ for people ages 60 and older; ABRYSVO^®^ for pregnant women at 32 to 36 weeks gestation; and Beyfortus^®^ for prevention in babies (2, 3). Despite such recent advances, the complex mechanisms by which RSV has evolved to evade the host immune response across its lifespan are not fully understood, and the approved products have received only limited endorsement by the U.S. Centers for Disease Control. The ongoing unmet medical need provides the focus of this Research Topic, titled *How RSV Outsmarts the Host*.

Frontiers in Immunology recently published four notable articles prepared by 59 authors from four countries. The topics covered were as follows: (i) “*Development and comparison of immunologic assays to detect primary RSV infections in infants*”, by Anderson et al.; (ii) “*Determinants of immunoglobulin G responses to respiratory syncytial virus and rhinovirus in children and adults*” by Guillien et al.; (iii) “*Lung ILC2s are activated in BALB/c mice born to immunized mothers despite complete protection against respiratory syncytial virus*”, by Kosanovich et al.; and (iv) “*Respiratory syncytial virus NS1 inhibits anti-viral Interferon-α-induced JAK/STAT signaling, by limiting the nuclear translocation of STAT1*” by Efstathiou et al.

RSV generally causes mild disease. In some individuals, however, particularly infants under 1 year of age, it can lead to severe symptoms. Since the immune system matures over the first year of life, understanding the complex interplay between maternal immune factors, viral exposure, and the maturing immune system requires robust assays to track RSV-specific immunity in infants. Anderson et al. describe seven such assays: four enzyme immunoassays (EIAs) measuring IgG titers to specific antigens (lysates of A and B subgroup-infected Hep-2 cells, recombinant F protein, and subgroup-specific G proteins), two assays for neutralizing activity against A and B subgroups, and an EliSpot assay for T-cell activation in response to subgroup lysates. All assays were reliable and resistant to common blood sample interfering substances. The EIAs against lysates and F protein were highly sensitive, detecting RSV exposure in all 44 sera collected from children 6 months post-RSV infection, whereas G protein assays were less sensitive, consistent with the F protein’s immunodominance. Neutralizing and EliSpot assays were positive in about half the sera. Surprisingly, the EliSpot assay was positive in 60% of the PBMC samples from RSV-naïve infants, likely reflecting maternal immune carryover.

Immune responses to RSV and rhinovirus (RV) differ between individuals, and to date, personal determinants of RSV- and RV-specific antibody responses remain unknown. Guillien et al. examined factors influencing RSV- and RV-specific IgG responses in 530 children and 1241 adults from the Epidemiological study on the Genetics and Environment of Asthma (EGEA) cohort. Older age was associated with higher RSV-specific IgG levels in both groups, with additional associations in adults for active—and to a lesser extent former—smoking and seasons other than summer. In contrast, RV-specific IgG levels increased with age during childhood but declined in adulthood. Female sex and lower body mass index (BMI) were associated with higher RV-specific IgG levels in both children and adults, with female sex consistently showing higher levels than male sex, particularly for RV-B. Active/former smoking and non-summer seasons were also linked to higher RV-specific IgG levels in adults. In children, RSV-specific and RV-specific IgG was not associated with season; furthermore, RSV-specific IgG was not associated with sex or BMI. These findings underscore how age, sex, BMI, smoking, and seasonal factors shape IgG responses to respiratory viruses, with distinct patterns in children versus adults.

Group 2 innate lymphoid cells (ILC2s) in the respiratory mucosa of human infants and mice play a key role in early RSV responses and subsequent adaptive Type 2 immunity, with elevated ILC2 levels linked to disease severity. Recent mouse studies showed that pups born to RSV-vaccinated dams, while protected from disease, possessed hyperresponsive ILC2s (hILC2). Kosanovich et al. investigated how hILC2 cells were activated during neonatal RSV infection in the presence of RSV-neutralizing maternal antibodies (matAb). They observed a non-statistical increase in ILC2 numbers and a significant rise in IL5+ILC2s frequency in the right lungs of RSV-infected pups from vaccination despite the absence of a replicating virus. ILC2 activation was driven by RSV-specific matAb: RSV immune complexes functioning through Fcγ receptors, which were differentially expressed on mouse and human respiratory ILC2s from immunized versus unimmunized dams. This study suggests maternal antibodies may not fully modulate all aspects of protective immune responses and highlights the need for further research on how maternal RSV vaccination influences infant immunity.

RSV employs multiple proteins to suppress the host’s innate immune response, with nonstructural proteins, NS1 and NS2, being particularly effective in inhibiting type I interferon (IFN) production and signaling, reducing immune cell recruitment and cytokine production. Efstathiou et al. show that RSV-NS1 enhances IFN-α-induced phosphorylation of STAT1 but reduces its nuclear translocation. Specifically, RSV-NS1 disrupts STAT1’s interaction with KPNA1, a nuclear import protein, as confirmed by protein-docking models. Because RSV-NS1 does not induce STAT2 phosphorylation, IFN simulated gene factor 3 (ISGF3) formation is impaired, further inhibiting STAT binding to gamma IFN activation sites (GAS) and IFN stimulated response element (ISRE) sites in antiviral gene promoters. It is important to note that these studies were performed in BEAS-2b cells, an immortalized human bronchial epithelial cell line, and that the role of young age in the lack of IFN-I responses, which are an important consideration for therapeutic strategies, was not evaluated.

The Research Topic *How RSV Outsmarts the Host* reveals a complex interplay between the virus and host immune responses. Development of therapeutics and vaccines has been hindered by limited understanding of age-dependent differences in immune responses and by gaps in knowledge of infants’ prior RSV infection status. By suppressing type I IFN production, manipulating mitochondrial functions, inducing autophagy, and modulating immune responses, RSV effectively evades host defenses and promotes its own replication. Gaining further insights into these mechanisms, armed with a better understanding of the infant’s immune state, is crucial for advancing antiviral strategies and vaccine development against RSV. However, more research is needed to fully unravel these complex interactions and to develop age-specific therapies that address the unique challenges of RSV infection in infants.
